# HCV-Induced Immunometabolic Crosstalk in a Triple-Cell Co-Culture Model Capable of Simulating Systemic Iron Homeostasis

**DOI:** 10.3390/cells10092251

**Published:** 2021-08-30

**Authors:** Pelagia Foka, Alexios Dimitriadis, Eirini Karamichali, Emmanouil Kochlios, Petros Eliadis, Vaia Valiakou, John Koskinas, Avgi Mamalaki, Urania Georgopoulou

**Affiliations:** 1 Molecular Virology Laboratory, Hellenic Pasteur Institute, 11521 Athens, Greece; eirinik@pasteur.gr (E.K.); kochlios.embio@gmail.com (E.K.); uraniag@pasteur.gr (U.G.); 2 Molecular Biology and Immunobiotechnology Laboratory, Hellenic Pasteur Institute, 11521 Athens, Greece; aldimitriadis@pasteur.gr (A.D.); peliadis@pasteur.gr (P.E.); vanessa.valiakou@gmail.com (V.V.); amamalaki@pasteur.gr (A.M.); 3 2nd Department of Internal Medicine, Hippokration Hospital, Medical School of Athens, 11527 Athens, Greece; koskinasj@yahoo.gr

**Keywords:** iron, hepcidin, HCV, innate immunity, cytokines, co-culture, ferritin, enterocytes, macrophages

## Abstract

Iron is crucial to the regulation of the host innate immune system and the outcome of many infections. Hepatitis C virus (HCV), one of the major viral human pathogens that depends on iron to complete its life cycle, is highly skilled in evading the immune system. This study presents the construction and validation of a physiologically relevant triple-cell co-culture model that was used to investigate the input of iron in HCV infection and the interplay between HCV, iron, and determinants of host innate immunity. We recorded the expression patterns of key proteins of iron homeostasis involved in iron import, export and storage and examined their relation to the iron regulatory hormone hepcidin in hepatocytes, enterocytes and macrophages in the presence and absence of HCV. We then assessed the transcriptional profiles of pro-inflammatory cytokines Interleukin-6 (IL-6) and interleukin-15 (IL-15) and anti-inflammatory interleukin-10 (IL-10) under normal or iron-depleted conditions and determined how these were affected by infection. Our data suggest the presence of a link between iron homeostasis and innate immunity unfolding among liver, intestine, and macrophages, which could participate in the deregulation of innate immune responses observed in early HCV infection. Coupled with iron-assisted enhanced viral propagation, such a mechanism may be important for the establishment of viral persistence and the ensuing chronic liver disease.

## 1. Introduction

Cellular well-being largely depends on iron needed for effective metabolism, energy production and building block biosynthesis [1]. Iron homeostasis is a tightly modulated process in the body. Following the absorption of ingested iron in duodenal enterocytes by the apical membrane divalent metal transporter-1 (DMT1), the iron molecules are either complexed by the iron storage protein ferritin in the cytoplasm or exported into circulation by the only known mammalian exporter Ferroportin (FPN). Then, the iron molecules are oxidised by ferroxidase hephaestin and bound anew by iron transporter transferrin (Tf). Plasma membrane transferrin receptor-1 (TfR1) will subsequently import iron into recipient cells through clathrin-mediated endocytosis, and DMT1 located onto the vesicles of the endocytic pathway releases iron molecules into the cytoplasm to be complexed anew by ferritin. The 25-amino-acid peptide hormone hepcidin is encoded by the hepcidin antimicrobial peptide (HAMP) gene and is mainly liver- and, to a lesser extent, macrophage-secreted. Hepcidin orchestrates systemic iron regulation by binding and degrading FPN, thereby inhibiting iron export and sequestering iron intracellularly [1,2,3,4]. Hepcidin itself is down-regulated by transmembrane serine protease matriptase-2 (TMPRSS6) via the suppression of hepcidin-inducing signalling [5,6]. Apart from enterocytes, macrophages and hepatocytes play key roles in systemic iron homeostasis, by serving as iron depositories. On top of that, macrophages contribute by recycling iron through the phagocytosis of aged erythrocytes [7,8]. 

Infectious microorganisms desperately need host iron for their survival [4,9]. Higher organisms have evolved efficient mechanisms to defend themselves against iron hijacking by invading pathogens. In this respect, the role of iron in innate immunity has been intensely scrutinised over the past years. Following infection, pro-inflammatory cytokines IL-6, interleukin-22 (IL-22) and interleukin-1 (IL-1) produced by cells of the innate immune system, such as natural killer (NK) cells and macrophages, initiate acute phase response in the liver. Consequently, the liver produces hepcidin among a plethora of innate immunity mediators [10,11,12]. The hepcidin-FPN-ferritin axis enables sequestration of iron in hepatocytes or macrophages, thereby blocking the access of dangerous extracellular siderophilic bacteria to iron [4]. On the other hand, other pathogenic bacteria have developed mechanisms to grab ferritin-bound iron and deploy countermeasures against host defences to fulfil their iron needs [1,13]. Evidently, chronic disorders and genetic conditions involving the deregulation of iron homeostasis may predispose to or protect against serious infectious diseases, such as malaria and tuberculosis [1,14]. 

Hepatitis C virus (HCV) is a positive-strand RNA virus of the *Flaviviridae* family, largely responsible for chronic hepatitis, liver cirrhosis and hepatocellular carcinoma (HCC). In most cases, HCV establishes persistent infection that goes undetected for decades. New effective therapies called direct acting antivirals (DAAs) eliminate the virus in most patients; however, they do not reduce HCC development risk, especially in patients with set-in fibrosis [15]. It has been suggested that iron imbalance predisposes to tumourigenesis directly, through mutagenic oxidative stress or indirectly through cancer-promoting signalling and immunometabolic alterations that steer the immune system towards tumour development tolerance [16]. Hepatic iron overload and hyperferraemia are a hallmark of hepatitis C [17,18] and have been strongly correlated to HCC development in chronic HCV patients [19,20].

Importantly, iron is crucial for the completion of the HCV life cycle, as it participates in HCV protein translation [21]. In addition, we showed that HCV increases cellular HAMP gene expression in infected hepatocytes cultured alone or in the presence of macrophages. The redistribution of iron between these cell types enhanced viral replication and rendered macrophages viral reservoirs [22]. In the light of this, we wondered whether the HCV-mediated manipulation of iron homeostasis instigates changes in innate immune responses that could favour viral persistence, thereby promoting liver disease progression. In order to better address this question, we set up a triple-cell co-culture model consisting of hepatocytes, macrophages and enterocytes that would be able to simulate systemic iron homeostasis in a more physiologically relevant manner.

## 2. Materials and Methods

### 2.1. Cellular Systems and Viral Infections

THP-1 human monocytes, derived from a patient with acute monocytic leukaemia, were cultured in RPMI 1640 supplemented with 10% (*v*/*v*) heat-inactivated foetal calf serum, 2 mM glutamine and 100 U/mL penicillin/streptomycin. They were terminally differentiated to macrophages with 0.16 μM phorbol 12-myristate 13-acetate (PMA) for 24 h. Huh7.5 human hepatoma cells were maintained in high-glucose DMEM supplemented as above, with the addition of non-essential amino acids. CaCO_2_ human colon carcinoma enterocytes were kept in low-glucose DMEM with 10 mM (*w*/*v*) HEPES and similar supplements to Huh7.5 cells. 

For the indirect triple-cell co-culture experiments, CaCO_2_ cells were seeded onto the underside of hanging inserts with 0.4 µm polyethylene terephthalate (PET) membranes (Greiner) for 16 h. Upon cell adhesion, the inserts were transferred into the wells of a 6-well plate and the cells were fed three times per week for 21 days, so that fully polarised monolayers would be acquired [23]. Then, terminally differentiated THP-1 macrophages were placed on the top surface of the hanging inserts and allowed to attach on the PET membranes for 1 h. The macrophage/enterocyte-containing inserts were fitted into 6-well plates that held permissive Huh7.5 hepatoma cells and infected with HCV JFH-1 infectious particles at a multiplicity of infection (MOI) of 1, as described elsewhere [24]. The double-cell co-cultures of Huh7.5 hepatoma and THP-1 macrophages were assembled as described above, omitting the CaCO_2_ enterocytes. The hepatoma:macrophage seeding ratio was 5:1, so that the co-cultures would simulate the non-inflamed liver environment [25]. The mock-infected cultures were compiled and treated similarly to the infected co-cultures. The mock-infected cell populations were harvested together with their infected counterparts to eliminate any experimental bias. When appropriate, the tissue culture medium was complemented with 100 µM desferrioxamine (DFO). All cell populations were harvested every 24 h for up to 96 h post-infection (p.i.) and used in subsequent expression analyses.

### 2.2. Expression Analyses

The parallel isolation of total RNA and proteins in undivided samples was performed with the dual NucleoSpin^®^ RNA/protein kit (Macherey-Nagel), as instructed by the manufacturer. The RNA fraction was subjected to RT-qPCR with gene-specific primers, and the results were analysed with the internal standard-curve method and normalised to 18S rRNA to provide relative mRNA expression. The primer pair used for the human IL-15 gene was IL-15F: 5′ CAT TTT GGG CTG TTT CAG TGC AGG 3′ and IL-15R: 5′ GTT GTT TGC TAG GAT GAT CAG ATT T 3′, while for the human HAMP, 18S rRNA, IL-6 and IL-10 genes, primers have been published elsewhere [26,27,28]. NS3 mRNA determination was carried out to monitor HCV replication levels, with primers that have been described before [28]. In all PCR experiments, the relative mRNA expression of the mock-infected controls (mean ± SD) was set as 100% or 1-fold, and the values of the corresponding infected samples were calculated as ratios of those. 

The protein fraction was quantified with a commercially available kit (Macherey-Nagel); 20–50 μg of whole cell extracts were used in immunoblotting, as published before [26], with the following antibodies: β-actin (MAB1501; Millipore), FPN (MTP11; Alpha Diagnostics), ferritin (A0133; Dako), TMPRSS6 (ab56180; Abcam), TfR1 (13-6800; Invitrogen) and NRAMP2/DMT1 (sc-30120; Santa Cruz). The presented gel photographs are representative results from the samples derived from the same experiment and processed in parallel. All RNA and protein analysis experiments were performed at least in triplicate. 

The home-made ELISA used to quantitate secreted hepcidin levels has been described before [29]. The assays were performed with pooled supernatants from below and above the hanging inserts of our co-culture models, which was repeated at least three times in quadruplicates. For both immunoblotting and ELISA analyses, the relative protein expression of each individual mock-infected control was set as 100%, and the values of the corresponding infected samples were calculated as a percentage of those. For the analysis of time-induced changes in the mock-infected co-cultures, the 24 h sample (RNA or protein expression) was set as a 100% control, and the values of all other time points were calculated as a ratio of this. 

### 2.3. Statistical Analysis

Pairwise comparisons of means between the mock-infected and HCV-infected samples or other pairs of samples described in individual figure legends were performed using Student’s or Welch’s *t*-test with *p*-values of ≤0.05 considered as statistically significant (*, *p*-value ≤ 0.05; **, *p*-value ≤ 0.005). 

## 3. Results

### 3.1. HCV Infection Modulates Systemic Iron Homeostasis in a Physiologically Relevant In Vitro Environment

**Figure 1:**
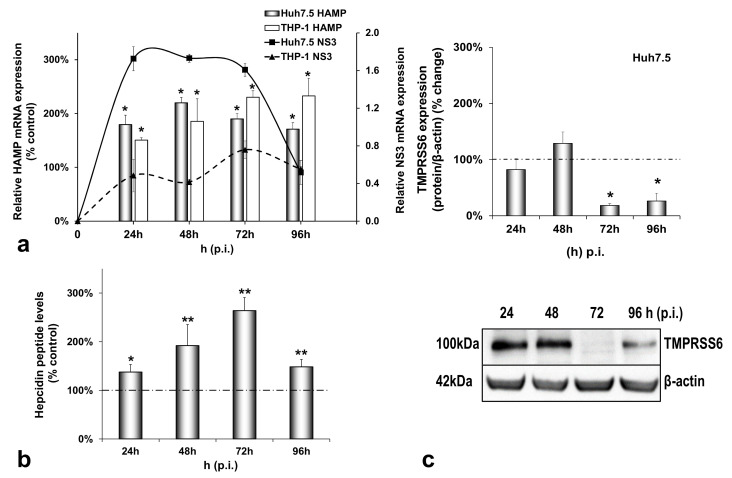
Differential regulation of hepcidin antimicrobial peptide (HAMP) and its inhibitor TMPRSS6 during hepatitis C virus (HCV) infection. HAMP and HCV NS3 mRNA expression (**a**) and hepcidin-secreted peptide levels (**b**) were measured in triple-cell co-cultures following infection with HCV JFH-1. RNA was isolated from Huh7.5 hepatoma cells and THP-1 macrophages and subjected to RT-qPCR at 24 h intervals post-infection (p.i.). 18S rRNA was used as an internal control. Secreted peptide concentrations were measured by ELISA in pooled supernatants. The mock-infected controls per time interval were arbitrarily set as 100% (represented by the dashed line), and all plotted values are the percentage of these. (**c**) TMPRSS6 was detected in Huh7.5 whole cell extracts from the same triple-cell co-cultures described in (**a**,**b**). Isolated protein was used in immunoblotting analysis, and β-actin was the loading control. The blots were subjected to densitometry and plotted in expression histograms. Values were calculated as a percentage of the respective mock-infected controls (represented by the dashed line). All graphs depict the average expression from at least three individual experiments, with representative accompanying blots. Statistical significance is denoted by asterisks as follows: *, *p*-value ≤ 0.05; **, *p*-value ≤ 0.005.

**Figure A1:**
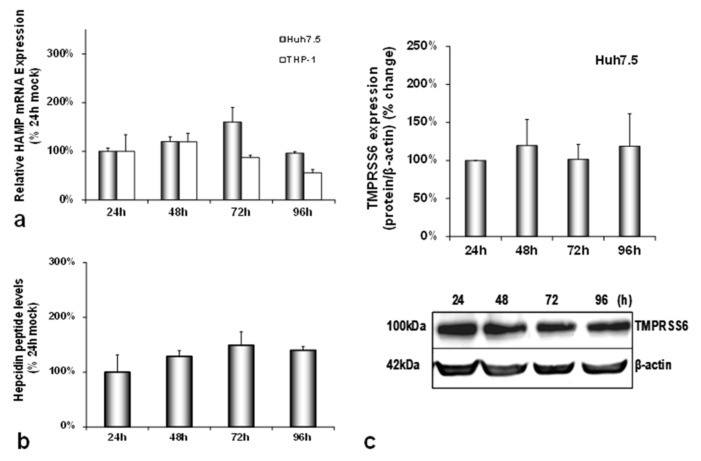
Basal gene expression of hepcidin and its inhibitor TMPRSS6 in triple-cell co-cultures. HAMP mRNA expression (**a**) and hepcidin-secreted peptide levels (**b**) were measured in mock-infected triple-cell co-cultures. RNA was isolated from Huh7.5 hepatoma cells and THP-1 macrophages and subjected to RT-qPCR at 24 h intervals. 18S rRNA was used as an internal control. Secreted peptide concentrations were measured by ELISA in pooled supernatants. The control of 24 h was arbitrarily set as 100%, and all plotted values are the percentage of this. (**c**) TMPRSS6 was detected in Huh7.5 whole cell extracts from the same triple-cell co-cultures described in (**a**,**b**). Isolated protein was used in immunoblotting analysis, and β-actin was the loading control. The blots were subjected to densitometry and plotted in expression histograms. Values were calculated as a percentage of the 24 h mock-infected control. All graphs depict the average expression from at least three individual experiments, with representative accompanying blots.

In order to investigate how the HCV-mediated modulation of systemic iron homeostasis could influence viral persistence and the innate immunity of the host, we created a more physiologically relevant in vitro environment for iron studies by co-culturing enterocytes with hepatocytes and macrophages. The triple-cell co-culture model was infected with the HCV JFH-1 laboratory clone for 96 h. For expression studies of iron-related proteins, the cells were harvested at 24 h intervals, used for RNA isolation and whole cell extract preparation which were subjected to RT-qPCR and Western blotting, respectively. The cell extracts were also utilised for the biochemical determination of intracellular iron. Finally, cell culture supernatants were collected for ELISA analysis. Figure 1a depicts a 1.5–2.5-fold increase in HAMP mRNA expression in both Huh7.5 and THP-1 cells over time, as compared to the mock-infected controls (Figure A1a). This increase was accompanied by a similar elevation in secreted hepcidin peptide in pooled co-culture supernatants (Figure 1b) that was not observed in supernatants from the mock-infected cells (Figure A1b). In fact, both mRNA and secreted hepcidin peptide expression remained quite stable when compared to the 24 h controls of the mock-infected co-cultures (Figure A1a,b). Thus, although moderate, the changes in hepcidin expression in the presence of the virus were enough to elicit sustained NS3 mRNA expression between 24 h and 72 h in hepatocytes and up to 96 h in macrophages (Figure 1a), denoting elongated HCV replication. To ascertain the replication benefits our triple-cell co-culture model offered to HCV, we measured HCV NS3 mRNA expression in Huh7.5 monocultures, Huh7.5/THP-1 co-cultures and the triple-cell model simultaneously. The Huh7.5 hepatoma cells (Supplementary Materials Figure S1a) and the THP-1 macrophages (Supplementary Materials Figure S1b) of the triple-cell co-culture model demonstrated the highest and most elongated mRNA expression among all tested populations. THP-1 monocultures did not survive when infected with HCV JFH-1 at an MOI of 1; therefore, they were not included in Supplementary Materials Figure S1b. Polarised CaCO_2_ monocultures have been reported to sustain very low levels of HCV replication [30]; however, we were unable to detect HCV NS3 mRNA in this cell population in our infected co-cultures. 

Interestingly, the observed HCV-mediated induction of hepcidin expression was accompanied by a profound decrease of its negative regulator matriptase-2 (TMPRSS6) by at least 80% towards the later stages of infection (Figure 1c), as compared to the mock-infected controls. As demonstrated in Figure A1c, the latter remained practically unaltered until the end of the experiment.

**Figure 2:**
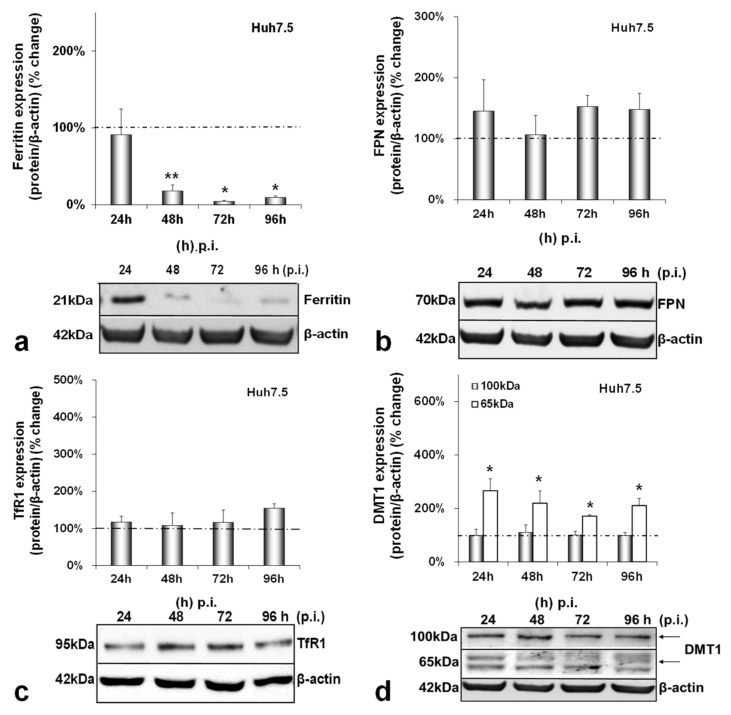
Differential regulation of hepatic iron-related proteins by HCV infection. Protein expression of ferritin (**a**), FPN (**b**), TfR1 (**c**) and DMT1 (100 kDa and 65 kDa isoforms) (**d**) were detected in Huh7.5 whole cell extracts from the same triple-cell co-cultures described in Figure 1. Isolated protein was used in immunoblotting analysis, and β-actin was the loading control. The blots were subjected to densitometry, and the resulting expression values were plotted in expression histograms. Values were calculated as a percentage of the respective mock-infected controls (represented by the dashed line). All graphs depict the average expression from at least three individual experiments, with representative accompanying blots. Statistical significance is denoted by asterisks as follows: *, *p*-value ≤ 0.05; **, *p*-value ≤ 0.005.

**Figure A2:**
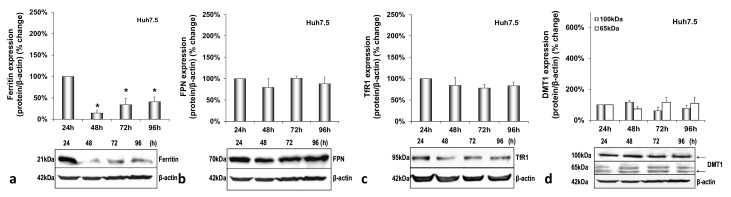
Basal hepatic protein expression in mock-infected triple-cell co-cultures. Ferritin (**a**), FPN (**b**), TfR1 (**c**) and DMT1 (100 kDa and 65 kDa isoforms) (**d**) polypeptides were detected in Huh7.5 whole cell extracts from Supplementary Materials Figure S1. Isolated protein was used in immunoblotting analysis, and β-actin was the loading control. The blots were subjected to densitometry, and the resulting expression values were plotted in expression histograms. Values were calculated as a percentage of the 24 h mock-infected control. All graphs depict average expression from at least three individual experiments, with representative accompanying blots. Statistical significance is denoted by asterisks as follows: *, *p*-value ≤ 0.05.

Next, we investigated the effect of HCV on iron homeostasis by monitoring the protein expression of ferritin (iron storage), FPN (iron export), TfR1 and DMT1 (iron import) over time in hepatocytes, macrophages and enterocytes. In infected hepatocytes, ferritin was dramatically reduced by 80–90% from 48 h p.i. until the end of infection (Figure 2a). On the other hand, FPN and TfR1 remained practically stable (Figure 2b,c). With respect to DMT1 expression levels, we observed the expression of two known isoforms in our blots, as others have described [31]. The non-glycosylated form of the protein (65 kDa) was up-regulated 2–3-folds throughout infection, while the glycosylated polypeptide (100 kDa) remained unchanged (Figure 2d). In the mock-infected co-cultures, ferritin was decreased by 60–80% between 48 and 96 h in cultures (Figure A2a), but there were no noticeable alterations in FPN, TfR1 and DMT1 isoforms (Figure A2b–d).

**Figure 3:**
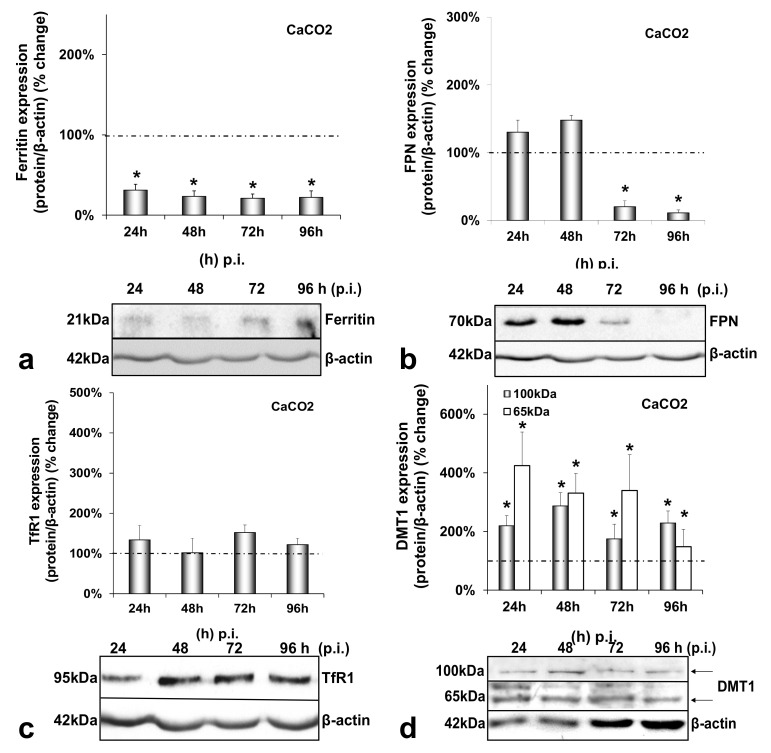
Differential regulation of intestinal iron-related proteins by HCV infection. Protein expression of ferritin (**a**), FPN (**b**), TfR1 (**c**) and DMT1 (100 kDa and 65 kDa isoforms) (**d**) were detected in CaCO_2_ whole cell extracts from the same triple-cell co-cultures described in Figure 1. Isolated protein was used in immunoblotting analysis and β-actin was the loading control. The blots were subjected to densitometry, and the resulting expression values were plotted in expression histograms. Values were calculated as a percentage of the respective mock-infected controls (represented by the dashed line). All graphs depict average expression from at least three individual experiments, with representative accompanying blots. Statistical significance is denoted by asterisks as follows: *, *p*-value ≤ 0.05.

**Figure A3:**
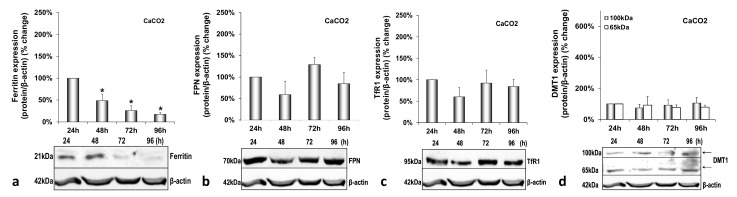
Basal intestinal protein expression in mock-infected triple-cell co-cultures. Ferritin (**a**), FPN (**b**), TfR1 (**c**) and DMT1 (100 kDa and 65 kDa isoforms) (**d**) polypeptides were detected in CaCO_2_ whole cell extracts from the same triple-cell co-cultures described in Supplementary Materials Figure S1. Isolated protein was used in immunoblotting analysis, and β-actin was the loading control. The blots were subjected to densitometry, and the resulting expression values were plotted in expression histograms. Values were calculated as a percentage of the 24 h mock-infected control. All graphs depict the average expression from at least three individual experiments with representative accompanying blots. Statistical significance is denoted by asterisks as follows: *, *p*-value ≤ 0.05.

CaCO_2_ enterocytes also suffered a dramatic reduction in ferritin levels reaching 70–80% throughout HCV infection (Figure 3a). Intestinal FPN was significantly down-regulated by 80–90% between 72 h and 96 h p.i. (Figure 3b), while TfR1 expression remained unchanged in both infected and mock-infected cells (Figure 3c and Figure A3c). Interestingly, DMT1 was found up-regulated during HCV infection, with a steady 2-fold increase for the 100 kDa isoform and a 4-fold elevation between 24 h and 96 h p.i. for the 65 kDa isoform, which gradually fell to a 1.5-fold elevation (Figure 3d). The changes in FPN and DMT1 were unique to the infected co-cultures and were not observed in the mock-infected enterocytes (Figure A3b,d). On the contrary, the ferritin expression showed a gradual decrease in the mock-infected cells, as observed in the mock-infected hepatocytes (Figure A3a).

**Figure 4:**
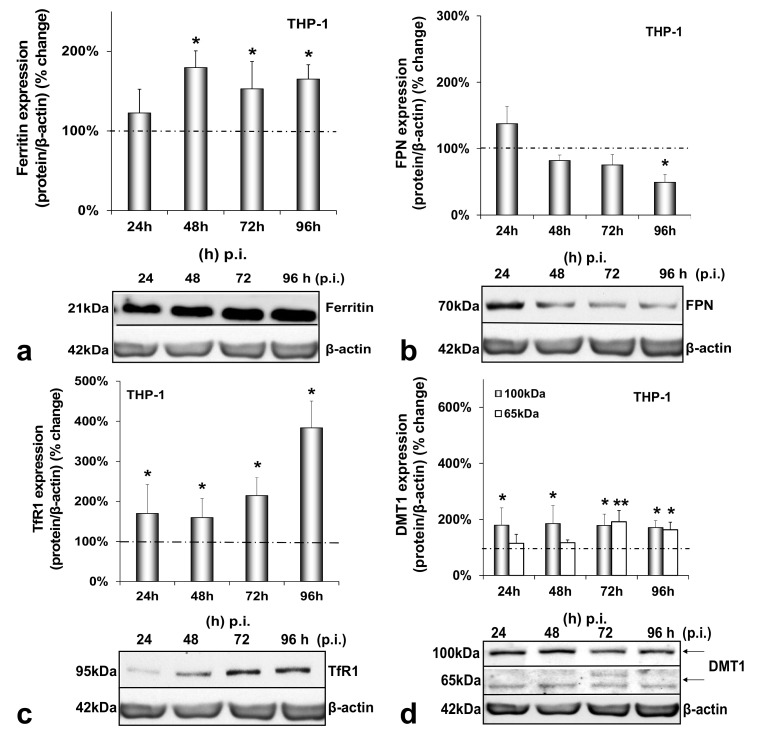
Differential regulation of macrophagic iron-related proteins by HCV infection. Protein expression of ferritin (**a**), FPN (**b**), TfR1 (**c**) and DMT1 (100 kDa and 65 kDa isoforms) (**d**) were detected in THP-1 whole cell extracts from the same triple-cell co-cultures described in Figure 1. Isolated protein was used in immunoblotting analysis, and β-actin was the loading control. The blots were subjected to densitometry, and the resulting expression values were plotted in expression histograms. Values were calculated as a percentage of the respective mock-infected controls (represented by the dashed line). All graphs depict average expression from at least three individual experiments, with representative accompanying blots. Statistical significance is denoted by asterisks as follows: *, *p*-value ≤ 0.05; **, *p*-value ≤ 0.005.

**Figure A4:**
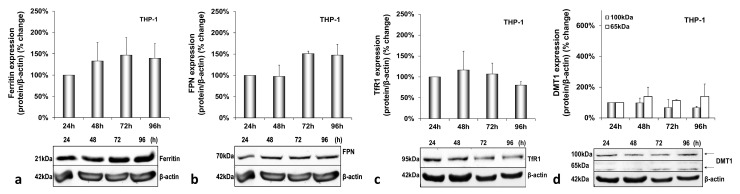
Basal macrophagic protein expression in mock-infected triple-cell co-cultures. Ferritin (**a**), FPN (**b**), TfR1 (**c**) and DMT1 (100 kDa and 65 kDa isoforms) (**d**) polypeptides were detected in THP-1 whole cell extracts from the same triple-cell co-cultures described in Supplementary Materials Figure S1. Isolated protein was used in immunoblotting analysis, and β-actin was the loading control. The blots were subjected to densitometry, and the resulting expression values were plotted in expression histograms. Values were calculated as a percentage of the 24 h mock-infected control. All graphs depict the average expression from at least three individual experiments with representative accompanying blots.

The final cell type examined was the HCV-infected THP-1 macrophage. While macrophagic ferritin gained in expression (1.5–1.8-fold from 48 h p.i. onwards) (Figure 4a), FPN was down-regulated by half at the end of the infection (Figure 4b). In addition, iron import seemed to increase greatly in macrophages, as both TfR1 and glycosylated DMT1 were increased dramatically (1.7–4-fold between 24 h and 96 h p.i. for the former and 2-fold throughout infection for the latter) (Figure 4c,d). On top of that, the 65 kDa DMT1 isoform was up-regulated 2-fold during the second half of the infection (Figure 4d). Again, no statistically significant changes occurred in any of these proteins with time in the mock-infected co-cultures (Figure A4a–d).

### 3.2. HCV-Mediated Changes on Iron Homeostasis Bestow an Anti-Inflammatory Phenotype on HCV-Infected Cells

**Figure 5:**
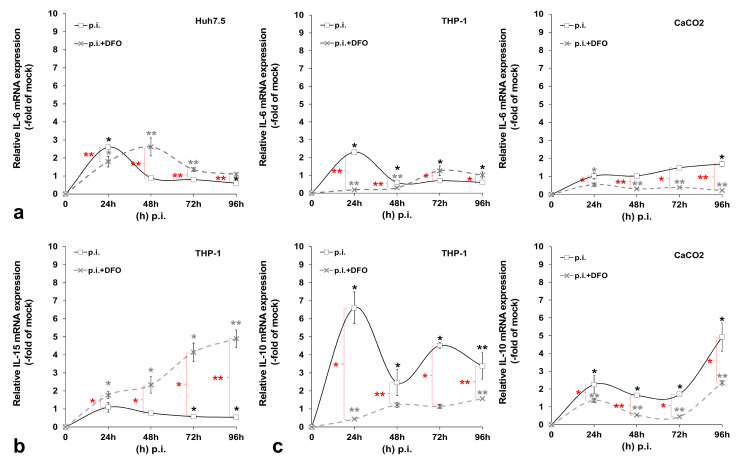
Pro- and anti-inflammatory cytokine expression profiling during HCV infection. The gene expression of pro-inflammatory interleukin (IL)-6 (**a**) and IL-15 (**b**), as well as anti-inflammatory IL-10 (**c**), was measured in cellular populations of the triple-cell co-cultures that are known to express the respective cytokine, following HCV infection in the presence or absence of desferrioxamine (DFO). mRNA expression levels were subjected to RT-qPCR with specific oligonucleotide primers. 18S rRNA was used as an internal control. The mock-infected controls per time interval were arbitrarily set as 1, and all plotted values are fold changes of these. All graphs depict the average expression of replicates acquired from at least three individual experiments. Statistical significance is denoted by asterisks as follows: *, *p*-value ≤ 0.05; **, *p*-value ≤ 0.005 (black asterisks: statistical significance of infected over the mock-infected controls; grey asterisks: statistical significance of the infected over mock-infected controls treated with DFO; red asterisks: statistical significance between the infected samples −/+ DFO).

**Figure A5:**
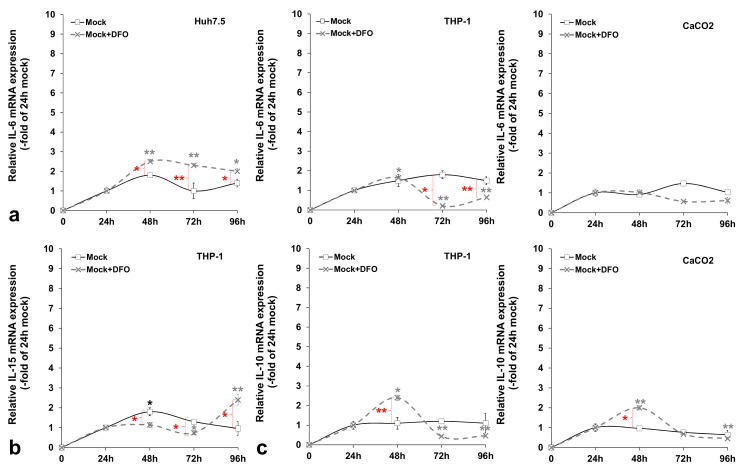
Basal pro- and anti-inflammatory cytokine expression profiling in mock-infected triple-cell co-cultures. The gene expression of pro-inflammatory IL-6 (**a**) and IL-15 (**b**), as well as anti-inflammatory IL-10 (**c**), was measured in cellular populations of the triple-cell co-cultures that are known to express the respective cytokine, in the presence or absence of DFO. mRNA expression levels were subjected to RT-qPCR with specific oligonucleotide primers. 18S rRNA was used as an internal control. The 24 h mock-infected control was arbitrarily set as 1, and all plotted values are fold- changes of this. All graphs depict the average expression of replicates acquired from at least three individual experiments. Statistical significance is denoted by asterisks as follows: *, *p*-value ≤ 0.05; **, *p*-value ≤ 0.005 (black asterisks: statistical significance of the infected over mock-infected controls; grey asterisks: statistical significance of the infected over mock-infected controls treated with DFO; red asterisks: statistical significance between the infected samples −/+ DFO).

Previous data from our laboratory and others have demonstrated that HCV blocks cytokine-mediated immune responses [22,32,33]. We wondered whether the HCV-mediated regulation of iron homeostasis could lead to the alteration of certain aspects of the innate immune system, thereby promoting a more tolerant cellular milieu for the establishment of HCV persistence. To test this hypothesis, we investigated expression patterns of pro-inflammatory (IL-6 and IL-15) and anti-inflammatory (IL-10) cytokines in the corresponding cells that produce them by RT-qPCR. Figure 5 shows cytokine mRNA levels calculated as folds of expression in HCV-infected over the mock-infected cells at 24 h intervals for a total of 96 h. IL-6 was increased by 2-fold at 24 h in both hepatocytes and macrophages and then dropped to mock-infected levels. There was a small (1.7-fold of the control) but significant IL-6 increase in enterocytes at 96 h p.i. Notably, upon the incubation of the infected cells with the iron chelator DFO, the IL-6 up-regulation was delayed by 24 h in Huh7.5 hepatocytes, while IL-6 expression was blocked in THP-1 cells for at least 48 h, whereupon it returned to background levels. CaCO_2_ IL-6 was down-regulated profoundly by DFO (ranging between 2.5- and 4-fold lower than that of control cells) (Figure 5a). In the mock-infected co-cultures, cytokine mRNA expression is depicted as the fold change of the 24 h sample. In this case, IL-6 mRNA expression was mostly increased after the first 24 h in cultured hepatocytes and macrophages (ranging between 1.4-fold and 1.8-fold) and remained at basal levels in CaCO_2_ cells. DFO seemed to affect some of these changes in a statistically significant manner, with the exception of enterocytes (Figure A5a). Macrophagic IL-15 was demonstrated to be down-regulated by 40–50% during the last two days of infection. This effect was counteracted by the presence of DFO, which induced IL-15 by 2–5-fold from beginning to end of infection (Figure 5b). In the mock-infected cells, we observed a 2-fold increase at 48 h, which was abolished by DFO (Figure A5b). 

Finally, we observed a dramatic elevation of IL-10 gene transcription in THP-1 macrophages throughout HCV infection that ranged from approximately 7-fold at 24 h to 4-fold at 96 h. The HCV effect was definitely reversed by DFO. Interestingly, CaCO_2_ enterocytes also showed a major gradual increase of IL-10 mRNA levels in HCV-infected co-cultures (up to 5-fold at 96 h p.i.). Incubating the cells with DFO dampened down this HCV-mediated effect (Figure 5c). In the mock-infected co-cultures, we could not detect any significant alterations in macrophagic or intestinal IL-10 mRNA expression with time. On the contrary, DFO mediated a 2-fold up-regulation in both cell lines at 48 h, which was then decreased to background levels and even reduced by half at the last day of culture (Figure A5c). It is possible that the milder changes recorded in the mock-infected cell, as opposed to HCV-directed gene transcription, could be attributed to cellular stress developed under the triple-cell co-culturing conditions.

## 4. Discussion

HCV, one of the major viral human pathogens that depends on iron to complete its life cycle [2], is highly skilled in evading the immune system [34,35]. The role of iron in the virus–host immune system relationship is not well-defined and constitutes the focus of the present study. We generated a triple-cell co-culture model containing hepatocytes, macrophages and enterocytes, to simulate systemic iron homeostasis. We used CaCO_2_ enterocytes, which recapitulate many functions of primary intestinal epithelial cells, upon spontaneous differentiation and polarisation on PET membranes [36]. Then, we determined the expression profiles of key iron-related proteins to verify that our model could reproduce known functions of systemic iron homeostasis and support viral infection. Finally, we investigated the role of iron in the HCV-mediated regulation of the host innate immune response.

We have previously described a positive regulation of HAMP gene expression by HCV in hepatoma/macrophage co-cultures [22], and the presence of enterocytes did not disturb this interaction (Figure 1a,b). We now report a profound down-regulation of hepcidin-negative regulator TMPRSS6 [5] (Figure 1c) towards the latter half of HCV infection. Low intracellular iron increases TMPRSS6 stability, and the iron chelator DFO up-regulates TMPRSS6 protein expression [37]. Although we observed a TMPRSS6 increase in DFO-treated hepatoma cells between 24 h and 48 h, HCV infection minimised TMPRSS6 expression altogether (Supplementary Materials Figure S2), indicating that HCV could overturn the positive impact exerted by iron deficiency on TMPRSS6. Furthermore, the transient elevation of known negative TMPRSS6 regulator IL-6 [38] between 24 h and 48 h p.i. (Figure 5a) could also contribute to the HCV-mediated decrease of TMPRSS6. The combination of increased hepcidin and reduced TMPRSS6 signalled enhanced capacity for iron sequestration in the infected hepatocytes. Still, the considerably low levels of hepatic ferritin, unaltered FPN, TfR1 and glycosylated DMT1 (Figure 2) contradicted this notion. The time-dependent decrease of ferritin could be attributed to its natural turnover rate [39]. Ferritin degradation is absolutely necessary for iron release and is inhibited by iron influx [39,40]. Iron which is not caged into ferritin molecules is shuttled intracellularly by iron chaperones, such as poly(rC)-binding protein-1 (PCBP1), an RNA-binding protein that acts as an iron donor to ferritin [41]. PCBP1 also binds to the HCV internal entry ribosome site (IRES) [42], which initiates HCV translation in an iron-dependent way within the first 24 h after entry [21]. Therefore, the HCV-induced reduction of hepatic ferritin could be due to virus-mediated degradation of this protein, so that intracellular iron may be channelled to viral translation. The intracellular bacterium *Neisseria meningitides* is known to utilise a similar mechanism for iron acquisition [43]. 

The noticeable increase of the 65 kDa isoform of DMT1 throughout infection also supports the idea of hepatic intracellular iron redirection towards viral translation. DMT1 exhibits differential cell- and iron-specific functions through strict multi-level regulation [31,44]. The 100 kDa isoform is mostly functional in polarised cells, such as macrophages and enterocytes [31], while the 65 kDa DMT1 isoform is involved in ferritin–iron mobilisation from the lysosomes to the cytoplasm after the TfR1-mediated entry of iron into the cell [45,46]. The second iron import receptor TfR1 remained unchanged in the HCV-infected hepatocytes of our model, despite having been shown to be down-regulated by HCV in a long-term infected monoculture of Huh7.5 cells [47]. Previous data from hepatoma monocultures and hepatoma/macrophage co-cultures [22] showed that an HCV-mediated increase in hepcidin expression resulted in reduced FPN towards the late hours of infection. Surprisingly, adding enterocytes in the infected co-culture somehow rendered the hepcidin-mediated down-regulation of hepatic FPN redundant. Animal studies justify this by demonstrating that hepatic FPN does not play an essential role in hepatic iron mobilisation, since iron needs may normally be met by macrophagic iron stores and enhanced intestinal absorption, the exception being cases of moderate-to-severe iron deficiency [48]. Importantly, hepatic iron measurements showed that, compared with the mock-infected cells, iron levels were at their highest at the start of the viral life cycle. There was also an iron decrease during the peak of HCV replication, which is in agreement with previous work from our group showing iron dependency of HCV replication (Supplementary Materials Table S1) [22].

Figure 3 demonstrates that, during HCV infection, CaCO_2_ ferritin was found down-regulated, TfR1 remained unchanged, and the 65 kDa isoform of DMT1 was up-regulated, similarly to their hepatic counterparts. The decrease of FPN during the second half of infection (Figure 3b) suggested that, in the triple-cell co-culture model, the hepatoma cells could sense the enterocytes and secreted hepatic hepcidin targeted intestinal ferroportin in order to reduce circulating iron. FPN has been shown to be down-regulated by hepcidin in vivo, while in vitro data with CaCO_2_ cells did not reflect such changes [49]. However, a recent study on the effect of hepcidin on intestinal iron absorption in chronic HCV patients demonstrated a reciprocal relationship between serum hepcidin and intestinal FPN [50]. Those authors agreed with our results that were not produced by CaCO_2_ monocultures, but a more physiologically relevant cellular environment. Furthermore, the 100 kDa glycosylated isoform was found increased in enterocytes. Several researchers reported a hepcidin-mediated decrease of DMT1 in vivo and in vitro in CaCO_2_ cells [49,51,52], so that hepcidin may block intestinal iron uptake and consequently reduce circulating iron. In our model, the hepcidin effect was bypassed in the presence of HCV. Iron measurements carried out in CaCO_2_ cells (Supplementary Materials Table S1) could not detect intracellular iron during the first 48 h that coincided with HCV translation and replication. Nevertheless, the degradation of FPN at 72 h p.i. combined with the increased DMT1 must have restored iron content in enterocytes from that time point onwards. Taken together, our results indicated that HCV most likely orchestrated iron efflux from CaCO_2_ cells at least during the crucial time frame for viral propagation. How iron was released into circulation under these circumstances is unclear.

Figure 4 demonstrates the expression of macrophagic iron-related proteins throughout HCV infection. In macrophages, ferritin, TfR1 and DMT1 isoforms were all up-regulated compared to controls, while FPN was down-regulated to keep iron stored inside the cells, as instructed by enhanced hepcidin [49,53,54]. Similarly, efficient transcription of human immunodeficiency retrovirus (HIV) has also been shown to be dependent on the iron equilibrium regulated by the hepcidin–FPN interplay [55]. Notably, ferritin may be released by hepatic cells through a non-canonical secretory pathway, its secretion cued in by incoming iron and pro-inflammatory cytokines [56,57,58]. A similar effect was noticed in HCV-infected hepatoma/macrophage co-cultures in previous studies from our laboratory [22]. Therefore, an HCV-directed ferritin secretion, assisted by transient hepatic IL-6 induction (Figure 5a), could partially account for the increased macrophagic ferritin. The enhanced TfR1 expression recorded in macrophages (Figure 4c) could also be involved in escorting ferritin in macrophages [59], since TfR1 acts as a ferritin importer in these cells [60]. In fact, the TfR1 increase may well be related to the HCV-mediated increase of IL-10 gene expression in the infected THP-1 cells (Figure 5c), since IL-10 has been shown to regulate TfR1 in activated monocytes, thereby favouring iron influx in these cells [61]. At the same time, increased DMT1 could enhance iron influx in the infected macrophages. A putative regulation of DMT1 by hepcidin may be a possibility in our model, as recent studies described the hepcidin-mediated up-regulation of DMT1 in macrophages in bacterial infections [62,63] and needs to be verified. Finally, the determination of macrophage iron recorded elevated iron levels with time as expected (Supplementary Materials Table S1).

In summary, we demonstrated that our model efficiently recapitulated the physiology of iron homeostasis in vitro and HCV deeply affected various aspects of iron homeostasis, from iron import to iron storage and export, effectively rerouting iron within and between cells in order to promote viral propagation. The enhanced and sustained NS3 expression in the infected hepatocytes and macrophages (Figure 1a and Supplementary Materials Figure S1), which far exceeded the typical NS3 climax previously observed in HCV infection in vitro by us and others [22,64], supports this notion.

As discussed above, some of the observed HCV-mediated changes in iron homeostasis proteins could be linked to altered cytokine levels in our model. The opposite may also be true, inasmuch as HCV could regulate major innate immunity constituents and curb initial anti-viral responses, by altering iron homeostasis. Both processes could be equally important for the successful infection and establishment of extrahepatic reservoirs and chronicity. Figure 5 depicts alterations in the transcriptional profiles of pro-inflammatory IL-6 and IL-15 and anti-inflammatory IL-10, recorded in our triple-cell co-culture model throughout the HCV infection. Most of these HCV-mediated changes were counteracted by DFO, thereby hinting at a link between iron homeostasis and the transcriptional regulation of these cytokines. Indeed, iron and iron-related proteins, such as hepcidin, FPN, TfR1 and ferritin, have been reported to induce signalling pathways that lead to the transcriptional regulation of pro- and anti-inflammatory cytokines, thereby modulating innate immune responses in infection and non-communicable diseases, such as cancer and non-alcoholic steatohepatitis [65,66,67,68,69]. 

Unsurprisingly, macrophages have been placed at the forefront of this immunometabolic battle when it comes to host immunity against infection and non-communicable diseases such as cancer [70,71,72]. Depending on host needs, macrophages mount either a pro-inflammatory M1 response able to kill intracellular pathogens or an anti-inflammatory, immunosuppressive M2 response. Cytokine gene expression could be a useful tool to assess the polarisation status of the infected macrophages. Evidently, the HCV-infected macrophages in our model are closer to assuming an M2 phenotype, with deeply decreased IL-15 and dramatically increased IL-10 gene expression. Other authors have also used the “IL-15/IL-10 pair” to characterise infected macrophages [73]. Iron may confer the M1 phenotype on macrophages implicated in inflammatory-based diseases, such as non-alcoholic fatty liver disease (NAFLD) [74] and atherosclerosis [75]. On the contrary, iron could tilt the equilibrium towards the inflammation-abating M2 phenotype, which promotes pathogen survival and persistence, as in the case of the intramacrophage bacterium *Salmonella typhimurium* [4]. By looking at a wide range of M1/M2 markers, of which the study falls out of the scope of this work, Kao and colleagues reported that THP-1 macrophages assumed an M2-like phenotype in an in vitro model of chronic iron overload, with increased IL-10 and almost undetectable IL-6 secretion [76]. The incubation of our triple-cell co-culture model with ferric ammonium citrate (FAC) for 4 days did not elicit such a response for IL-6 and IL-10 transcription in the presence of iron alone, but only in combination with HCV infection (data not shown). This could be due to the presence of the other cell populations that may have influenced macrophage polarisation in our model, thereby suggesting that such immune responses must be coordinated by many factors in vivo. Indeed, several studies highlight the key role of the liver and intestine in the immune responses through cytokine production that aims to recruit professional immune cells to the sites of infection and injury [11,77,78,79]. The opposite also stands true with polarised macrophages being able to drive iron homeostasis and dictate infection outcomes for the host [80]. Thus, HCV may establish a vicious cycle that progressively inhibits a successful innate immune response by the host. This may have serious connotations for viral elimination during early infection, since it has been suggested that the iron-mediated deregulation of innate immune responses may in turn affect adaptive immune responses, possibly on top of a more direct effect of iron-driven changes on T-lymphocytes [70]. The profound down-regulation of the T-lymphocyte inducer IL-15 [81] throughout early HCV infection in macrophages could provide a hint towards such a response, as the reduced expression of IL-15 could result in blunted T-cell responses later in infection. 

## 5. Conclusions

**Figure 6:**
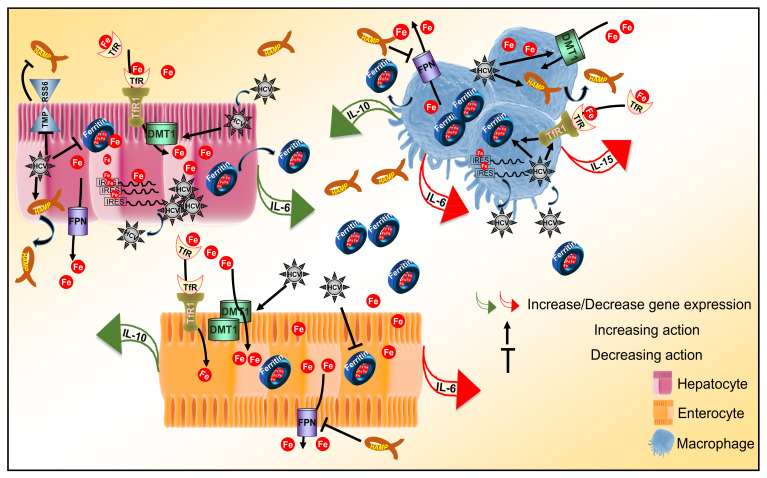
Iron homeostasis and innate immunity determinants in HCV infection. Schematic diagram of the immunometabolic interplay between HCV, iron and determinants of the innate immune response in a triple-cell co-culture model. The diagram schematically captures major findings reported in this study by depicting transcriptional alterations of cytokine genes and translational expression changes of iron homeostasis proteins elicited by HCV infection.

In conclusion, we constructed a more physiologically relevant cellular system for the study of iron homeostasis that proved able to truthfully capture several aspects of iron biology. The intricate dynamics unfolding between our cellular model and HCV infection allowed us to record temporal cell-specific transcriptional changes of key pro- and anti-inflammatory cytokines under normal or iron-depleted conditions, as demonstrated in Figure 6. Therefore, our work may have unravelled a tangible link between iron homeostasis and these innate immunity determinants, which could be the basis for the “stalling” of innate immune responses in HCV infection. Coupled with iron-assisted enhanced viral propagation, such a mechanism may be important for the establishment of viral persistence and chronic liver disease with all its repercussions in host health and well-being.

## Supplementary Materials

The following are available online at www.mdpi.com/article/10.3390/cells10092251/s1, Figure S1: The triple-cell co-culture model promotes HCV replication, Figure S2: Attenuation of TMPRSS6 protein expression by HCV in the presence and absence of iron chelation by DFO. Table S1: Iron measurements in the triple-cell co-culture system during HCV infection, Supplementary Methods: Iron measurements.
