# Supplementary material for: HCV-Induced Immunometabolic Crosstalk in a Triple-Cell Co-Culture Model Capable of Simulating Systemic Iron Homeostasis
Source: Cells. 2021 Aug 30;10(9):2251. doi: 10.3390/cells10092251 (PMC8465420; doi:10.3390/cells10092251)
Supplement: Supplementary file 1 [file cells-10-02251-s001.zip › cells-1315961-supplementary/Supplementary -Foka et al/Supplementary Table Foka et al.pdf]

Supplementary Table S1. Iron measurements in the triple-cell co-culture system during HCV infection.

Cell line	(h) p.i.	Fe Concentration (ng/µl) ± SD	-fold change of mock-infected control
Huh7.5	24	1.63 ± 0.16	1.4
	48	0.72 ± 0.07	0.6
	72	1.10 ± 0.11	0.9
	96	1.08 ± 0.10	0.9
CaCO2	24	b.d.l.	-
	48	b.d.l.	-
	72	2.33 ± 0.23	1.7
	96	1.06 ± 0.11	0.8
THP-1	24	1.10 ± 0.11	0.9
	48	1.32 ± 0.13	1.1
	72	1.78 ± 0.18	1.5
	96	1.85 ± 0.19	1.6

Key: SD, Standard deviation; b.d.l., below detection limit.

Supplementary Materials and Methods

Iron measurements

Whole cell extracts from Huh7.5, CaCO2 and THP-1 cells from HCV- and mock-infected triple-cell co-cultures were prepared as described in Materials and Methods, diluted 1:10 with phosphate buffer saline (PBS) and subjected to iron measurements using an Abbott ARCHITECT 2000 biochemical analyzer, in triplicate.
