# Supplementary figures and images for: HCV-Induced Immunometabolic Crosstalk in a Triple-Cell Co-Culture Model Capable of Simulating Systemic Iron Homeostasis
Source: Cells. 2021 Aug 30;10(9):2251. doi: 10.3390/cells10092251 (PMC8465420; doi:10.3390/cells10092251)

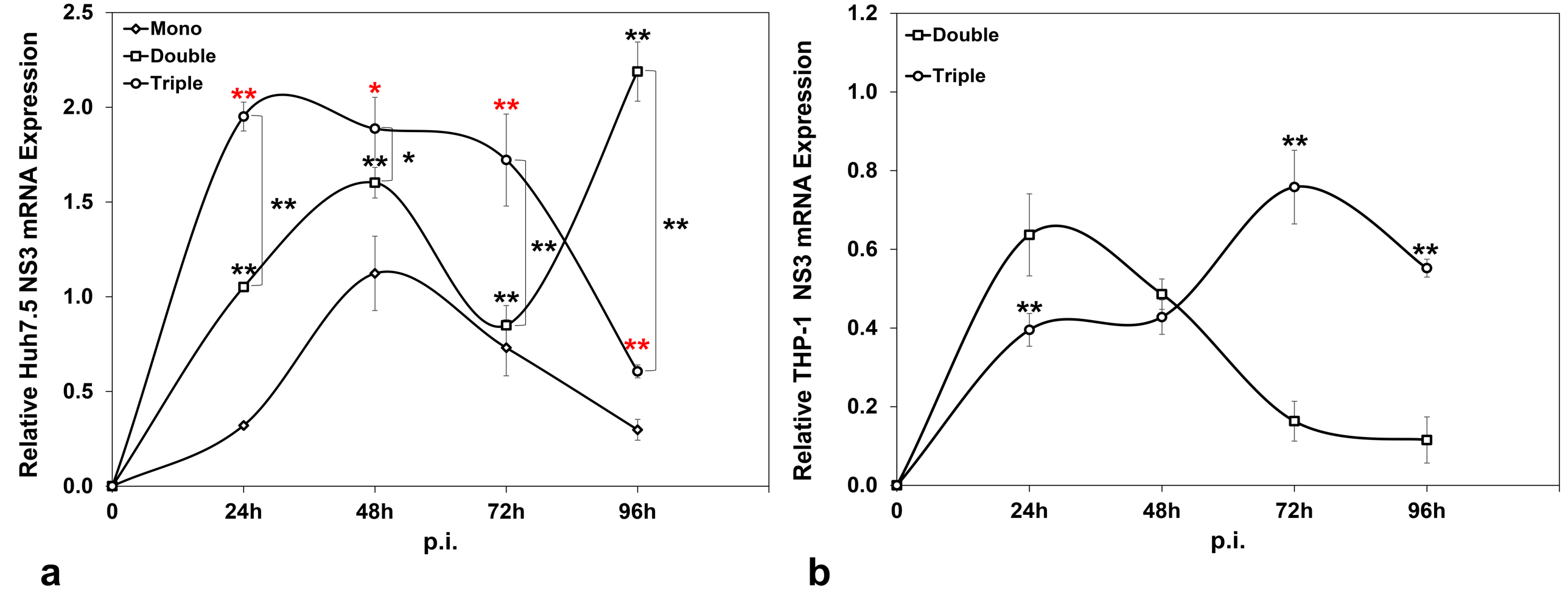

Supplement: Supplementary file 1 [file cells-10-02251-s001.zip › cells-1315961-supplementary/Supplementary -Foka et al/SUpplementary Figure S1.tif]

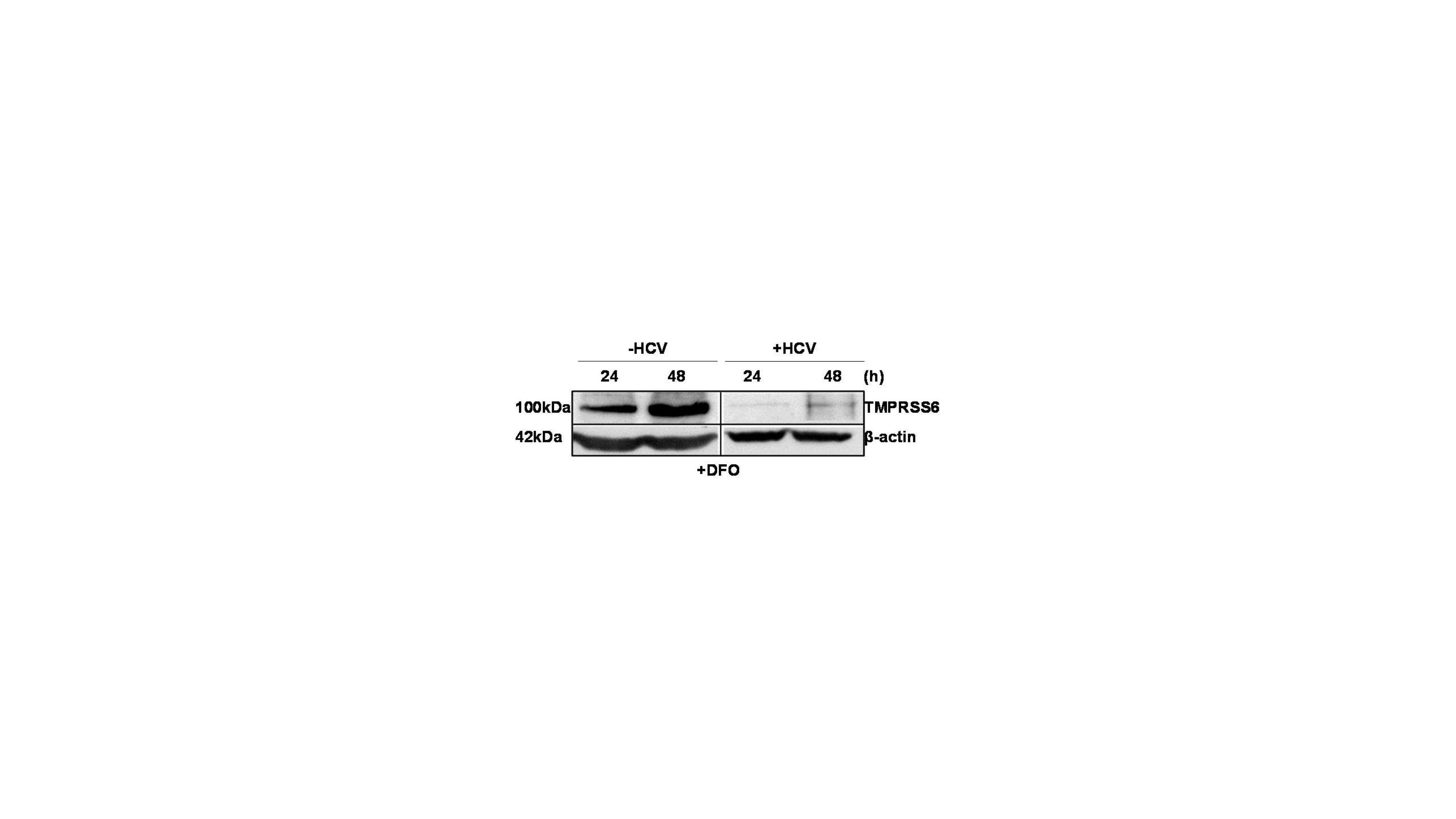

Supplement: Supplementary file 1 [file cells-10-02251-s001.zip › cells-1315961-supplementary/Supplementary -Foka et al/Supplementary Figure S2.tif]
